# A Missed Diagnosis of Intraprosthetic Dislocation in a Dual-Mobility Bearing Following Closed Reduction

**DOI:** 10.7759/cureus.49361

**Published:** 2023-11-24

**Authors:** Travis Ballard, Andrew Corbett, John D Murphy, William Judson, John N Harker

**Affiliations:** 1 Orthopedic Surgery, Largo Medical Center, Largo, USA

**Keywords:** dual-mobility bearings, dual mobility, total hip athroplasty, hip dislocations, intraprosthetic dislocation

## Abstract

Dual-mobility bearings have been found to reduce the rate of dislocation following both primary and revision total hip arthroplasty. Their unique design involves two articulating surfaces which increases construct stability but also leaves them susceptible to a unique complication known as intraprosthetic dislocation (IPD). We report the case of a 33-year-old female who sustained an IPD following closed reduction. Following a missed radiographic diagnosis, the patient experienced pain and mechanical symptoms secondary to her implant failure. Surgical removal of the dislodged liner with component revision was required. This case highlights several crucial steps in the management of patients with dislocated total hip arthroplasties including implant identification and careful review of postreduction radiographs. We also discuss several strategies to properly diagnose, manage, and avoid IPD.

## Introduction

Instability is one of the leading causes of revision surgery following total hip arthroplasty (THA) [1]. Several modifications to the conventional THA design are available to address this problem including dual mobility (DM) bearings. First introduced in the 1970s, DM bearings have been shown to reduce the frequency of postoperative hip dislocation in both primary and revision THA [2,3]. Given their enhanced stability, DM bearings are primarily chosen for patients at high risk of dislocation such as those with neuromuscular disease, spinal deformities, and hip dysplasia [4,5]. 

DM bearings have a unique design featuring a mobile polyethylene liner between the femoral head and acetabular shell, allowing for two points of articulation within the construct [6]. A small articulation between the prosthetic head and the polyethylene liner acts as the inner bearing and is the primary site of movement within the construct. A second, larger articulation between the polyethylene liner and acetabular shell acts as the outer bearing and only moves during extremes in range of motion. The additional articulation allows the construct to act as a larger diameter head which increases the jump distance, head-neck ratio, and ultimately stability [6-8]. 

A complication unique to DM bearings is intraprosthetic dislocation (IPD) which occurs when the polyethylene liner disengages from the prosthetic head [2,3,5,9]. In modern DM implants, IPD is most often an iatrogenic complication occurring during a closed reduction attempt for a dislocated outer articulation [10]. We report a case of a 33-year-old female who sustained an IPD following an attempted closed reduction which was initially missed on postreduction radiographs. This case highlights the importance of both implant identification prior to attempted THA reduction as well as careful reading of postreduction radiographs. We also discuss several strategies to properly diagnose, manage, and avoid IPD. 

## Case presentation

**Figure 1:**
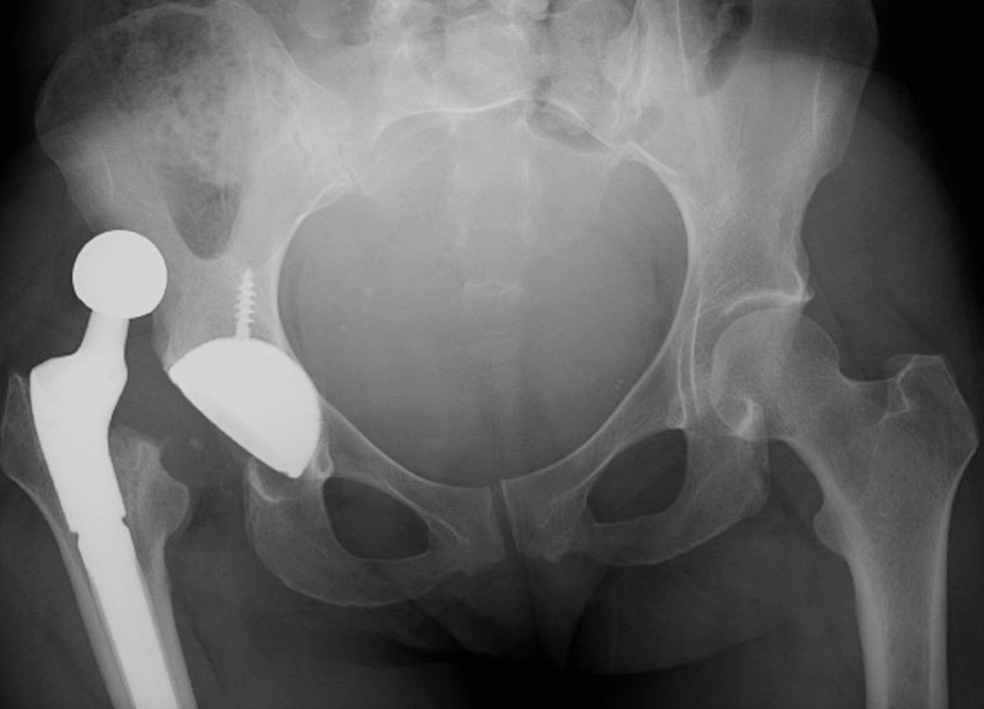
Prereduction Radiograph of the Pelvis AP view of the pelvis demonstrating a posterior dislocation of right total hip arthroplasty. AP: Anteroposterior

A 33-year-old white female with a history of avascular necrosis and septic arthritis of the right hip requiring two-stage revision arthroplasty presented to the emergency department with a dislocated right THA. She underwent conversion from a cemented antibiotic spacer to a dual mobility THA three months prior with components including a 50 mm acetabular cup, a 40 mm dual mobility polyethylene liner, and a 28 mm head ball. Initial radiographs confirmed a posterior dislocation of the total hip prosthesis (Figure 1). 

**Figure 2:**
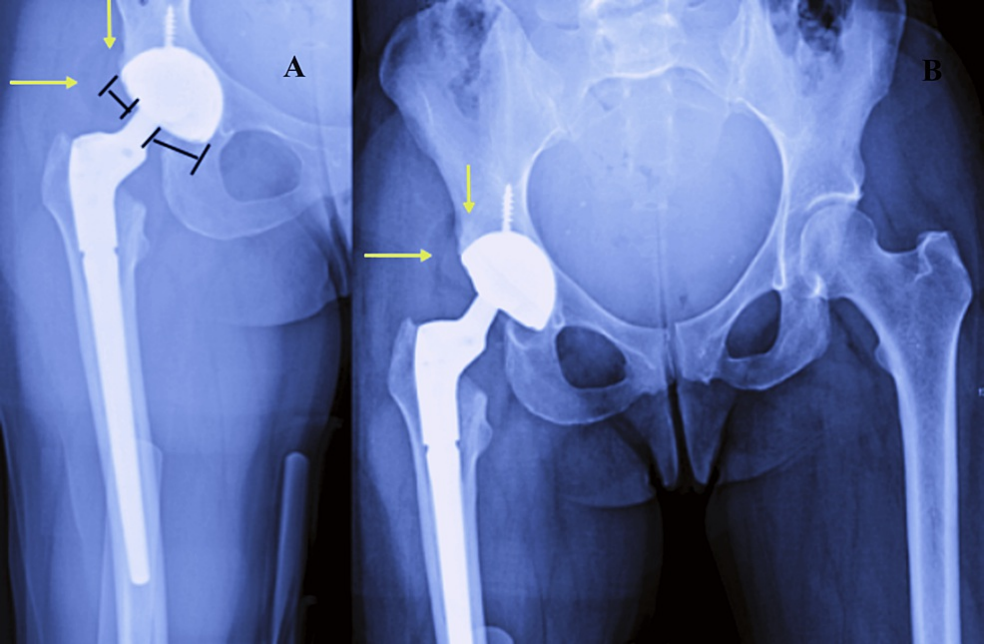
Postreduction Radiographs of the Pelvis and Right Hip A and B: AP views of the pelvis and right hip following closed reduction demonstrate eccentricity of the femoral head (black lines) within the acetabular shell. There is also a ring-shaped radiolucency (yellow arrows) representing the dislocated polyethylene liner (i.e., Bubble Sign). Both findings are indicative of an intraprosthetic dislocation. AP: Anteroposterior

Closed reduction under sedation was performed and postreduction radiographs were obtained (Figure 2). 

**Figure 3:**
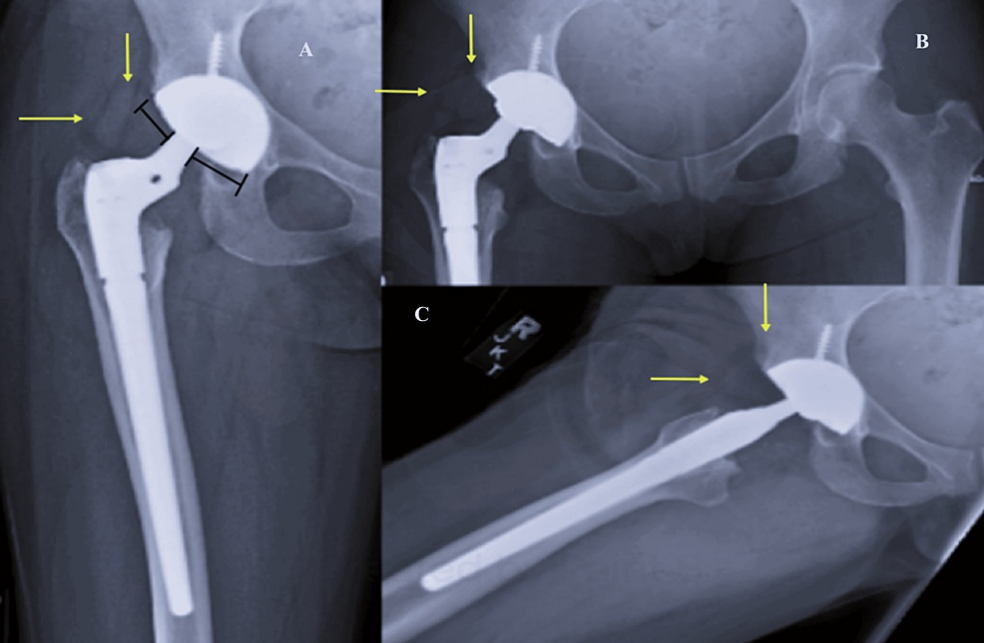
Postreduction Radiographs of the Pelvis and Right Three Weeks Following Closed Reduction A-C: AP pelvis, AP right hip, and lateral views of the right hip again demonstrate signs of intraprosthetic dislocation including eccentricity of the femoral head (black lines) as well as the ring-shaped radiolucency (yellow arrows) (i.e., Bubble Sign). AP: Anteroposterior

The reduction was deemed acceptable and she was discharged with instructions to weight bear as tolerated. The patient presented for follow-up three weeks later reporting pain in her right groin and thigh as well as experiencing a clicking sensation in her right hip with ambulation since discharge from the hospital. The postreduction radiographs were re-examined and showed eccentricity of the femoral head and a ring-shaped radiolucent object located superolateral to the hip joint. New radiographs were obtained and demonstrated similar findings (Figure 3). After comparing the pre- and postreduction radiographs, it was determined that an intraprosthetic dislocation occurred following the closed reduction attempt.

**Figure 4:**
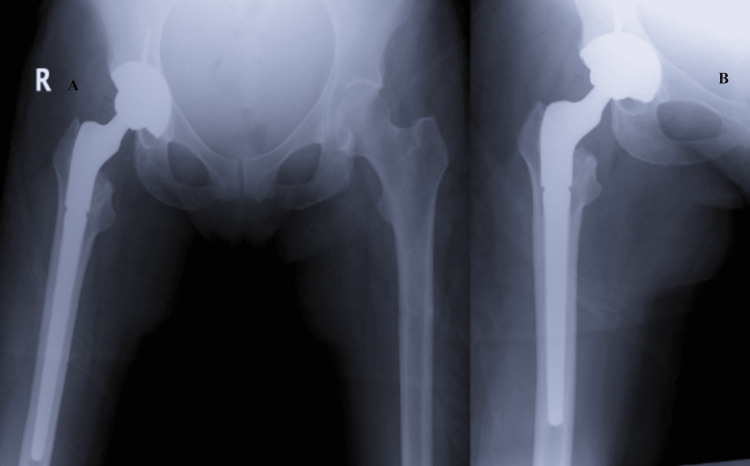
Radiographs of the Pelvis and Right Following Revision Arthroplasty A and B: AP pelvis and AP right hip following revision arthroplasty. Components are in proper alignment and position with no signs of loosening. AP: Anteroposterior

Three weeks later, the patient underwent revision surgery where the polyethylene liner was found superolateral to the hip joint, confirming the diagnosis of IPD. It was removed with a Kocher clamp and replaced with a liner of the same size. The acetabular cup was examined and found to be in acceptable alignment with no evidence of significant liner wear so it was left in place. Lastly, the femoral head was removed and replaced with a component of the same size. Postoperative radiographs were obtained and demonstrated proper alignment and positioning of all components (Figure 4). She was discharged the same day and allowed to weight bear as tolerated with instructions to maintain posterior hip precautions and use an abduction pillow while sleeping. She was seen in the clinic three weeks later where she reported no complaints and radiographs showed no signs of complication. 

## Discussion

Intraprosthetic dislocation is a relatively rare phenomenon with an incidence of 1.1% in primary THAs and 0.3% in revision THAs [1]. Historically, IPD was attributed to premature wear of the components; however, updated neck designs, the use of larger heads, liner-retentive rim modifications, and higher resistance polyethylene have drastically reduced this means of failure in modern designs [9,11,12]. A systematic review looking at outcomes of DM bearings in THA reported 0 cases of IPD in patients who underwent DM THA after 2007 [11]. The primary cause of IPD in modern designs is iatrogenic dislocation following closed reduction, evidenced by a recent systematic review which found that iatrogenic IPD was responsible for 79% of reported cases [9]. Iatrogenic IPD is most commonly seen following an attempted closed reduction for a dislocated outer bearing, with a recent case series noting an incidence rate of 71% [10]. The underlying mechanism is termed the "bottle-opener" effect and involves engagement of the polyethylene liner with the acetabular shell or bony prominence followed by disassociation of the liner during a subsequent reduction attempt [9,13]. 

The diagnosis of IPD begins with a proper history and physical exam where patients often report pain, clicking, or grinding within the hip joint [14]. Additional concerning symptoms include feelings of a foreign body within the hip and audible clicking sounds with ambulation. Radiographs of the hip should be obtained and scrutinized for findings suggestive of IPD. The “Bubble Sign”, a halo-shaped radiolucency near the components representing the dissociated polyethylene liner, is considered pathognomonic [15]. This radiolucency can be difficult to appreciate on conventional radiographs as it is often subtle or obscured by surrounding structures. A more reliable finding is the eccentric positioning of the femoral head within the acetabular shell. If IPD is suspected but radiographs are inconclusive, computed tomography (CT) can be used to reliably identify and locate a dislodged polyethylene liner. It is most often entrapped in the soft tissues surrounding the hip joint; however, intrapelvic migration is also possible [16]. 

Given the high rate of IPD following closed reduction, many authors suggest that reduction be obtained in the operating room with complete relaxation using spinal or general anesthesia [9,10]. Unlike conventional THA implants, DM bearings are difficult to reduce under conscious sedation in the ER as considerable forces are required to disengage the polyethylene liner from the acetabular shell. Complete patient relaxation in the operating room allows for the use of gentle reduction maneuvers thereby reducing the chances of the “bottle opener” effect. As no reduction maneuver for dislocated DM THA has been established, it is also recommended to utilize intraoperative fluoroscopy to aid with reduction. 

IPD warrants open reduction as retrieval and replacement of the dislodged liner are required. Operative treatment begins with an open exploration to locate and revise the polyethylene component [17]. If not located in the hip joint, intrapelvic migration of the liner should be suspected and identified with a postoperative CT scan. An intrapelvic liner can be left in place if found to not be compressing any vital structures [16]. The femoral head and acetabular components should then be assessed for signs of wear and stability. Any evidence of malalignment or significant wear warrants component revision. Surgery should be performed urgently in order to minimize the amount of time that the femoral head and acetabular shell articulate with one another. Friction between these two surfaces leads to increased rates of wear and may result in soft tissue metallosis and elevated cobalt and chromium levels [18,19]. 

In order to prevent this complication, providers should be familiar with the various types of DM implants and be able to identify them on radiographs. Two commonly used design options in the United States are anatomic dual mobility (ADM) systems and modular dual mobility (MDM) systems [10]. The outer articulation for the ADM is composed of the polyethylene liner and a cobalt chrome shell while the outer articulation for the modular systems features the polyethylene liner and a cobalt chrome liner seated within a titanium shell. Radiographically, ADM systems can be identified by an anterior recess on the rim to accommodate the iliopsoas tendon and a characteristic, nonhemispheric appearance owing to a deepening of the socket on the posteroinferior aspect [9,20]. 

## Conclusions

Iatrogenic dissociation following closed reduction is the primary mechanism underlying IPD in modern DM bearings. It is imperative to identify this implant design so that safe reduction may be performed under complete relaxation in the operating room. Characteristic radiographic findings of intraprostatic dissociation include eccentric positioning of the femoral head and the "Bubble Sign". Management of this complication should include urgent operative intervention to revise the polyethylene liner and any other damaged or malaligned implants. 
